# Safety and Efficacy of Oxaliplatin Pressurized Intraperitoneal Aerosolized Chemotherapy (PIPAC) in Colorectal and Appendiceal Cancer with Peritoneal Metastases: Results of a Multicenter Phase I Trial in the USA

**DOI:** 10.1245/s10434-023-13941-2

**Published:** 2023-07-27

**Authors:** Mustafa Raoof, Richard L. Whelan, Kevin M. Sullivan, Christopher Ruel, Paul H. Frankel, Sarah E. Cole, Raechelle Tinsley, Melissa Eng, Marwan Fakih, Joseph Chao, Dean Lim, Yanghee Woo, Isaac Benjamin Paz, Michael Lew, Michaela Cristea, Lorna Rodriguez-Rodriguez, Yuman Fong, Rebecca Meera Thomas, Sue Chang, Danielle Deperalta, Amit Merchea, Thanh H. Dellinger

**Affiliations:** 1 Department of Surgery, City of Hope National Medical Center, Duarte, CA USA; 2 Department of Surgery, Northwell Health, New York, NY USA; 3 Department of Computation and Quantitative Medicine, City of Hope National Medical Center, Duarte, CA USA; 4 Department of Clinical Protocol Development, City of Hope National Medical Center, Duarte, CA USA; 5 Clinical Trials Office, City of Hope National Medical Center, Duarte, CA USA; 6 Department of Medical Oncology and Therapeutics Research, City of Hope National Medical Center, Duarte, CA USA; 7 Department of Anesthesiology, City of Hope National Medical Center, Duarte, CA USA; 8 Department of Pathology, Norwell Health, New York, NY USA; 9 Department of Pathology, City of Hope National Medical Center, Duarte, CA USA; 10 Department of Surgery, Mayo Clinic, Jacksonville, FL USA

## Abstract

**Background:**

Pressurized intraperitoneal aerosolized chemotherapy (PIPAC) is a laparoscopic locoregional treatment for peritoneal metastases (PM) from colorectal cancer (CRC) or appendiceal cancer (AC) in patients who cannot undergo cytoreductive surgery (CRS). While PIPAC has been studied in Europe and Asia, it has not been investigated in the USA.

**Patients and Methods:**

We evaluated PIPAC with 90 mg/m^2^ oxaliplatin alone (cycle 1) and preceded by systemic chemotherapy with fluorouracil (5-FU) and leucovorin (LV) (cycle 2–3) as a multicenter prospective phase I clinical trial (NCT04329494). The primary endpoint was treatment-related adverse events (AEs). Secondary endpoints included survival and laparoscopic, histologic, and radiographic response.

**Results:**

12 patients were included: 8 with CRC and 4 with AC. Median prior chemotherapy cycles was 2 (interquartile range (IQR) 2–3). All patients were refractory to systemic oxaliplatin-based chemotherapy. Median peritoneal carcinomatosis index (PCI) was 28 (IQR 19–32). Six (50%) of twelve patients completed three PIPAC cycles. No surgical complications or dose-limiting toxicities were observed. Two patients developed grade 3 treatment-related toxicities (one abdominal pain and one anemia). Median overall survival (OS) was 12.0 months, and median progression-free survival (PFS) was 2.9 months. OS was correlated with stable disease by Response Evaluation Criteria in Solid Tumors (RECIST) criteria but not with laparoscopic response by PCI or histologic response by peritoneal regression grading system (PRGS).

**Conclusions:**

This phase I trial in the USA demonstrated safety, feasibility, and early efficacy signal of PIPAC with oxaliplatin and chemotherapy in patients with PM from AC or CRC who are refractory to standard lines of systemic chemotherapy.

**Supplementary Information:**

The online version contains supplementary material available at 10.1245/s10434-023-13941-2.

Peritoneal metastases (PM) from colorectal cancer (CRC) and appendiceal cancer (AC) impact on morbidity with malignant bowel obstruction, ascites, or obstructive uropathy, and have a median overall survival (OS) of 14 months, worse than either isolated lung or liver metastases.^1^ A proportion of these patients with more limited disease can undergo cytoreductive surgery (CRS) with or without hyperthermic intraperitoneal chemotherapy (HIPEC) and improved median OS of 41 months,^2^ but additional treatments are needed for patients with more advanced disease.

Pressurized intraperitoneal aerosolized chemotherapy (PIPAC) is an intraperitoneal drug delivery modality administered via a minimally invasive laparoscopic approach that can be repeated. Pressurization and aerosolization were demonstrated in preclinical models to homogenously distribute chemotherapy throughout the abdominal cavity with good tissue penetration.^3–5^ Oxaliplatin at a dose of 92 mg/m^2^ in PIPAC was initially demonstrated safe in a multicenter retrospective study,^6^ wherein 251 treatments were carried out in 101 patients with colorectal, gastric, ovarian, or other malignancies, with 15.9% of patients experiencing a Common Terminology Criteria for Adverse Events (CTCAE) grade IV or higher toxicity. The PIPOX trial (NCT03294252)^7^ was a phase I/II trial in France with a specific aim to determine the maximum tolerated dose (MTD) of oxaliplatin in PIPAC, starting at 90 mg/m^2^ with planned escalation by 50 mg/m^2^ up to 300 mg/m^2^ using a 3+3 design. In that study, ten patients with gastric, small bowel, or CRC underwent 33 PIPAC procedures with 11 grade III/IV toxicities that were mostly hematologic, abdominal (pain or nausea/vomiting), and fatigue. A MTD of 90 mg/m^2^ was determined.^8^ The PIPAC-OX trial (NCT03172416),^9^ performed in Singapore, was another phase I study aiming to determine the recommended phase II dose (R2PD) of oxaliplatin in PIPAC; 16 patients with mostly gastric cancer and CRC underwent 24 PIPAC procedures, and the R2PD dose determined was 120 mg/m^2^. However, three patients developed pancreatitis, two occurring at 45 mg/m^2^ and one at 90 mg/m^2^ dose of oxaliplatin. For eight patients who underwent two PIPAC procedures, peritoneal carcinomatosis index (PCI) and peritoneal regression grading score (PRGS) were reduced in this study. Finally, Robella et al.^10^ in Italy performed a phase I oxaliplatin PIPAC trial (NCT02604784) and recommended a higher dose of 135 mg/m^2^, with three patients who were treated at 135 mg/m^2^ experiencing grade I or II abdominal pain. As such, in current consensus guidelines for PIPAC, most experts favored an oxaliplatin dose of 90–92 mg/m^2^.^11^ A retrospective study of a prospective cohort of 30 patients with CRC PM who underwent oxaliplatin PIPAC in conjunction with 5-fluorouracil (5-FU) and leucovorin (LV) systemic chemotherapy demonstrated no difference in adverse events with oxaliplatin PIPAC plus 5-FU and LV compared with oxaliplatin PIPAC alone.^12^

Given these early clinical data from Europe and Asia indicating the overall safety of PIPAC with some efficacy in a challenging PM patient population who have progressed on previous lines of therapy and with limited alternative options, we sought to evaluate this novel therapy in the USA. While the prior studies in Europe and Asia include PM from gastrointestinal malignancies included gastric cancer, the focus of this trial was CRC and AC. Secondly, prior studies have included patients with heterogeneous exposure to prior systemic therapy. Here, we focused on a chemotherapy-refractory population. The primary aim of the study was to confirm the safety, feasibility, and pharmacokinetics of the previously studied dose and schedule of oxaliplatin PIPAC in combination with intravenous (IV) 5-FU and LV. Secondary aims included measures of clinical efficacy including OS, progression-free survival (PFS), response rate by laparoscopic PCI, radiographic response by Response Evaluation Criteria in Solid Tumors (RECIST) version 1.1, and histological response by PRGS. Lastly, to inform the design of future randomized phase II efficacy trial, we aimed to evaluate which surrogate markers of response correlate best with overall survival.

## Methods

### Ethics Statement

This study was conducted according to the principles of the Belmont Report: Ethical Principles and Guidelines for the Protection of Human Subjects or Research and the Declaration of Helsinki. All patients completed documented informed consent to participate. This study was approved by the City of Hope Institutional Review Board (IRB) (no. 19184), the Northwell Health IRB (no. 20-0859), and the Mayo Clinic IRB (no. 20-010121) and is being conducted under an open US multicenter phase I trial (NCT04329494). Specifically, the present study reports results from arm 2. Results from other arms will be reported as they mature.

### Patients

Adult patients ≥ 18 years old with histologically confirmed invasive AC or CRC with PM who had progressed on at least one previous standard chemotherapeutic treatment were included if they had Eastern Cooperative Oncology Group (ECOG) performance status (PS) ≤ 2, no contraindications for laparoscopy, and ≤ 5 L of ascites and were not a candidate for cytoreduction and/or HIPEC. Exclusion criteria were low-grade appendiceal mucinous neoplasm (LAMN) or high-grade appendiceal mucinous neoplasm (HAMN), bowel obstruction requiring nasogastric or gastrostomy tube or total parenteral nutrition, life expectancy less than 6 months, neutropenia with absolute neutrophil count (ANC) < 1500/mm^3^, thrombocytopenia with platelets < 100,000/mm^3^, previous anaphylactic reaction to any of the chemotherapy drugs used, simultaneous tumor debulking or gastrointestinal resection, or active ongoing infection or severe comorbidities. Previous intraperitoneal therapy was not an exclusion criterion.

### Study Design

This is a phase I nonrandomized and uncontrolled single-arm clinical trial without dose escalation to establish the safety of oxaliplatin PIPAC in cycle 1, and the combination of oxaliplatin PIPAC with 5-FU/LV in cycles 2–3 given within 24 h of the PIPAC. The rules for accrual were based on limiting the risk to that of the traditional 3+3 phase I trial design with modifications that allow for reduced time to complete the study without compromising patient risk.^13^ These rules are based on not exceeding the risks associated with the traditional 3+3 phase I design, while better accounting for the patient queue including the potential for inevaluable patients (patients who do not get fully treated or followed for reasons other than unacceptable treatment-related adverse event such as rapidly progressive disease). This approach has been used in several NCI studies (NCI CTEP protocols nos. 9091 and 9924, to reduce study duration without adversely impacting operating characteristics or patient safety). Unless the trial stops accrual for unacceptable AEs, the expected number of patients starting treatment is eight during the safety evaluation, which allows for inevaluable patients and a maximum of eight evaluable patients. Additional patients are permitted as noted above after the safety evaluation, up to 14 patients. The study required no more than one unacceptable treatment related AE (DLT) in the first six patients and no more than two in the first seven or eight patients. Once initial safety was established without unacceptable adverse events on the basis of those rules or any treatment death, the study planned for accrual of up to 14 patients. The PIPAC procedure was performed as previously described,^14^ with intraperitoneal drug delivered using a high-pressure injection (Medrad Stellant injector, Bayer Corporation) and Capnopen nebulizer (Capnomed Corporation, Tubingen, Germany) at a maximum of 200 psi and 30 mL/min, followed by a 30-min pneumoperitoneum at 12 mmHg containing the aerosolized at room temperature prior to release of the pneumoperitoneum. Oxaliplatin 90 mg/m^2^ was administered intraperitoneally (IP) via PIPAC at 6-week intervals for a total of three treatments provided no adverse event (AE), dose-limiting toxicity, disease progression, or patient withdrawal occurred. Prior to the second and third treatments, 5-FU 400 mg/m^2^ IV with LV 20 mg/m^2^ IV were administered for sensitization. There were no planned dose modifications for this study. If a patient was unable to receive the next scheduled PIPAC due to toxicity, a delay of up to 21 days was permissible, with any delay greater than 21 days considered a DLT. Other toxicities considered a DLT were any grade 3 or higher nonhematologic toxicity, excluding grade 3 nausea, vomiting, abdominal pain or diarrhea adequately treated to grade 2 or lower within 48 h; grade 3 fatigue that returns to grade 2 or less within 7 days; grade 3 laboratory/metabolic abnormalities that are not considered clinically significant and are easily correctable to grade 2 or lower within 72 h; grade 3 infusion-related reaction (first occurrence and in the absence of steroid prophylaxis) that resolves within 6 h with appropriate clinical management, and grade 3 peripheral neuropathy. Additionally, surgical complications of Clavien–Dindo grade IIIB or higher; Grade 4 thrombocytopenia, grade 4 neutropenia lasting more than 7 days or associated with fever or infection were also considered a DLT. The DLT rules were applied for cycle 2 to assess the safety of PIPAC oxaliplatin, and separately in cycle 2 to assess the addition of 5-FU/LV preceding PIPAC oxaliplatin.

### Endpoints

The primary objective is to evaluate the safety of PIPAC and is based on treatment-related AEs reported by the NCI CTCAE version 5.0. AEs were assessed every 4–6 weeks.

Secondary objectives included evaluation of progression-free survival, postoperative complication rate and response by imaging, PCI on laparoscopy, or histologic changes. Postoperative complications were graded according to Clavien–Dindo classification and evaluated 4 weeks after each PIPAC procedure. Response evaluation was based on RECIST version 1.1 criteria by imaging as a change from baseline at 10 weeks and 18 weeks. PCI determination was made at the time of each laparoscopy, with complete response (CR) defined as PCI < 3 with negative histology on at least three peritoneal biopsies, partial response (PR) defined as PCI decreased by 4, and progressive disease (PD) defined as PCI increased by 4. PRGS was evaluated by multiple biopsies performed at each laparoscopy for PIPAC procedure.

### Statistical Analysis

Survival analysis was assessed using the method of Kaplan and Meier. Measurements of association used a two-sided Fisher’s exact test. Statistical significance was defined at 0.05. Statistical analyses were performed using SAS.

## Results

### Patient Characteristics

**Fig. 1:**
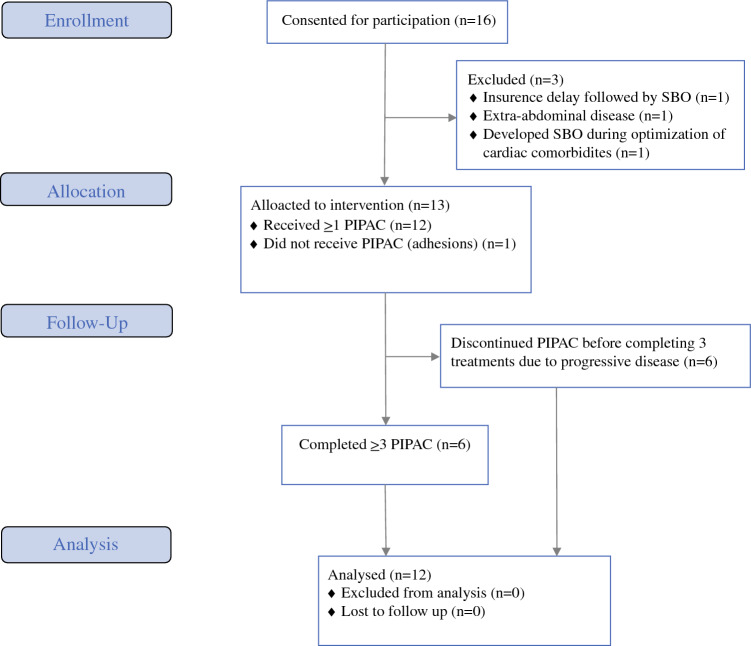
Consolidated Standards of Reporting Trials (CONSORT) flow diagram of the progression of patients through the trial, including enrollment, allocation, follow-up, and analysis. *SBO* small bowel obstruction

A total of 12 patients with CRC or AC received at least one PIPAC from August 2020 to January 2022 across the three sites in the USA (Fig. 1). The median age of patients was 60 years, and 42% of the patients were female. Eight (67%) patients had CRC, and the remaining 33% had AC. All had good performance status with Eastern Cooperative Oncology Group (ECOG) scores 0–1. The median PCI of patients was 28, and the median number of previous chemotherapy lines to which they were refractory was 2. Five (42%) patients had undergone a previous CRS, with or without HIPEC. These and other baseline characteristics of the 12 patients enrolled in the study are listed in Table 1.

**Table 1:** Demographics and characteristics of patients enrolled

Characteristic	*N* = 12
Median age in years (range)	60 (29–75)
Gender	
Male	7 (58%)
Female	5 (42%)
Race/ethnicity	
Asian	1 (8%)
Hispanic	1 (8%)
Non-Hispanic White	8 (67%)
Pacific Islander	1 (8%)
Undisclosed	1 (8%)
Performance status (ECOG)	
0	8 (67%)
1	4 (33%)
Site	
Appendiceal	4 (33%)
Colon	6 (50%)
Rectal/rectosigmoid	2 (17%)
Tumor characteristics	
Differentiation	
Well	2 (17%)
Moderate	4 (33%)
Poor	6 (50%)
Mucinous histology	9 (75%)
Signet ring cell	3 (25%)
*KRAS* mutant	5 (42%)
*BRAF* mutant	0 (0%)
Microsatellite stable	12 (100%)
Low TMB	12 (100%)
Median PCI (IQR)	28 (19, 32)
Median days from diagnosis to PIPAC (IQR)	476 (309, 560)
Median prior lines of chemotherapy (IQR)	2 (2, 3)
Prior radiation	
Yes	2 (17%)
No	10 (83%)
Prior cytoreduction with or without HIPEC	
Yes	5 (42%)
No	7 (58%)

*ECOG* Eastern Cooperative Oncology Group, *PCI* peritoneal carcinomatosis index, *IQR* interquartile range, *HIPEC* hyperthermic intraperitoneal chemotherapy, *TMB* tumor mutational burden

### Feasibility of PIPAC

There were no technical failures in completing the laparoscopy. One patient initially enrolled underwent laparoscopy but had prohibitive adhesions for PIPAC (Fig. 1). Median hospital length of stay following each PIPAC procedure was 1 day. Seven patients (58%) completed two or more PIPAC procedures, and six patients (50%) completed at least three PIPAC procedures. Median follow-up for the study was 5.4 months. Only disease progression prevented the second or third PIPAC treatments.

### Safety of PIPAC

There were no surgical complications by Clavien–Dindo classification, nor any DLT. Seven patients (58%) experienced grade I toxicities, four patients (33%) experienced grade II toxicities, and two patients (17%) experienced grade III toxicities. The most common toxicities were gastrointestinal, and the majority were grade I/II consisting of abdominal pain, nausea, vomiting, constipation, abdominal distention, and diarrhea. The most common toxicity overall was abdominal pain, which occurred in five patients (42%). Of the grade III AEs, one patient experienced abdominal pain and another experienced anemia (at least partially attributed to 5-FU bolus). There were no grade IV toxicities. A summary of all AEs is listed in Table 2. PIPAC oxaliplatin toxicities in cycle 1 were then compared with the toxicities associated with the second cycle of treatment with PIPAC oxaliplatin and systemic 5-FU/LV toxicities in cycle 2. These results, summarized in Supplementary Table 1, do not demonstrate any meaningful differences between the two groups.

**Table 2:** Adverse events during the treatment period

Type	Adverse event related to treatment	Grade 1	Grade 2	Grade 3
Constitutional	Fatigue	1 (8%)	1 (8%)	
	Hypotension		1 (8%)	
	Dizziness	1 (8%)		
	Generalized muscle weakness	1 (8%)		
	Muscle cramping	1 (8%)		
	Noncardiac chest pain	1 (8%)		
	Urine output decreased	1 (8%)		
	Anorexia	2 (17%)		
Gastrointestinal	Abdominal pain	3 (25%)	1 (8%)	1 (8%)
	Constipation	3 (25%)	1 (8%)	
	Nausea/vomiting	3 (33%)	1 (8%)	
	Ileus		1 (8%)	
	Abdominal distention	2 (17%)		
	Diarrhea	2 (17%)		
Hematologic	Anemia	1 (8%)		1 (8%)
	Thrombocytopenia		1 (8%)	
	Leukopenia	1 (8%)		
Electrolyte abnormality	Hypophosphatemia		1 (8%)	
	Hypernatremia	1 (8%)		
	Hypoalbuminemia	1 (8%)		
	Hypocalcemia	1 (8%)		
	Hyponatremia	1 (8%)		
	Hypokalemia	1 (8%)		

### Pharmacokinetics

**Fig. 2:**
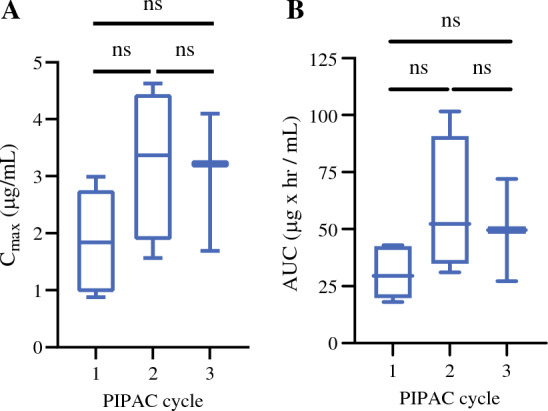
Pharmacokinetic data of serum maximum concentration (*C*_max_) and area under curve (AUC) following each PIPAC cycle. *Ns* not statistically significant

Pharmacokinetic data were available for six patients. Across all PIPAC procedures, the average oxaliplatin plasma maximum concentration (*C*_max_) was 2.55 (standard deviation (SD) 1.24) µg/mL with an average area under curve (AUC) of 43.97 (SD 23.41 µg × h/mL). The average *C*_max_ was 1.87 µg/mL in cycle 1, 3.23 µg/mL for cycle 2, and 3.00 µg/mL for cycle 3, which were not statistically significantly different (*p* > 0.1; Fig. 2A; Supplementary Table 2). Similarly, the average AUC was 30.51 µg × h/mL for cycle 1, 59.38 µg × h/mL for cycle 2, and 50.35 µg × h/mL for cycle 3, which were also not statistically significantly different (*p* > 0.1; Fig. 2B; Supplementary Table 2). Tissue oxaliplatin concentrations from both normal and tumor peritoneal biopsies taken immediately after PIPAC administration were 22.4 ng/mg for PIPAC 1, 14.5 ng/mg following PIPAC 2, and 24.5 ng/mg after PIPAC 3 (*p* > 0.05).

### Efficacy of PIPAC

**Fig. 3:**
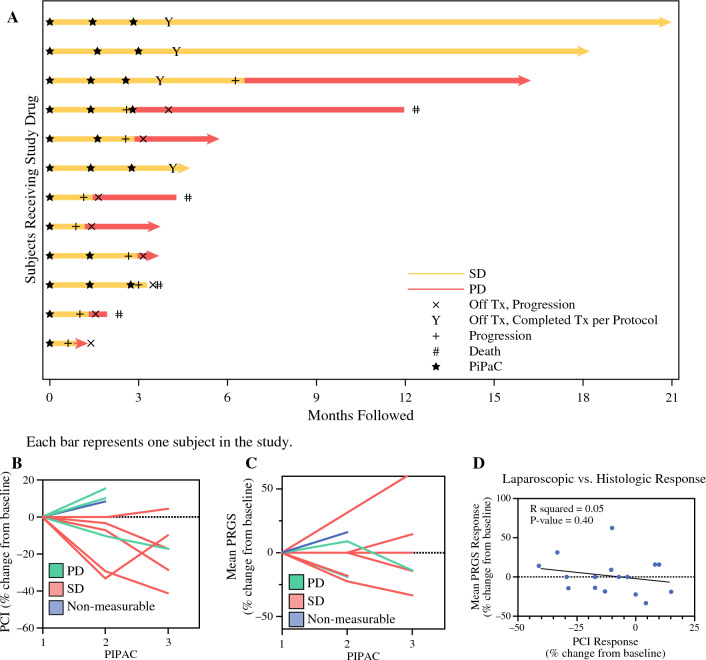
Response to PIPAC treatments. **A** Swimmer plot of each patient and response to treatment measured by imaging using RECIST criteria over time. Patients with stable disease are denoted with yellow bars, and progressive disease in red. **B** Depiction of change in laparoscopic PCI over each PIPAC cycle, with green lines demarking patients experiencing progressive disease by imaging RECIST criteria and red lines demarking patients with stable disease by imaging. **C** Depiction of change in histologic response by mean PRGS over each PIPAC cycle, with green lines demarking patients experiencing progressive disease by imaging RECIST criteria and red lines demarking patients with stable disease by imaging. **D** Change in PCI response compared with change in mean PRGS, demonstrating no association of PCI with PRGS. *RECIST* Response Evaluation Criteria in Solid Tumors, *PCI* peritoneal carcinomatosis index, *PRGS* peritoneal regression grading system

Response to PIPAC was assessed in several ways: (1) By radiographic RECIST version 1.1 criteria at best overall response, six patients (50%) demonstrated SD for ≥ 3 months, while another six (50%) had PD (Table 3; Fig. 3A); (2) By laparoscopic assessment at the time of second laparoscopy, six patients (50%) had a decrease in PCI score and the remaining had progressive disease or did not have laparoscopy owing to clinical progression (Table 3); and (3) By mean PRGS, histologic response or stable disease was seen in 7/12 (58%) patients (Table 3; Supplementary Table 3). All patients except one who had SD by radiographic evaluation had a decrease in PCI compared with baseline, including one patient with radiographic PD. Most patients who had a reduction in PCI after PIPAC continued to show decreased PCI score with subsequent treatments (Fig. 3B). The association between radiographic evaluation by RECIST and laparoscopic PCI was statistically significant (*p* < 0.01). Reductions in mean PRGS were correlated to SD by radiographic response (Fig. 3C). Similarly, changes in PRGS also did not correlate with changes in laparoscopic PCI (Fig. 3D).

**Table 3:** Response to oxaliplatin PIPAC with systemic therapy

	Best response	*N* = 12
Conversion to CRS-HIPEC	Yes	2 (17%)
	No	10 (83%)
Radiographic (RECIST)	SD	6 (50%)
	PD	6 (50%)
Laparoscopic (PCI)	Decrease	6 (50%)
	PD or only one PIPAC	6 (50%)
Histologic (PRGS, mean)	Decrease	5 (42%)
	SD	2 (17%)
	PD or only one PIPAC	5 (42%)

*CRS-HIPEC* cytoreductive surgery and hyperthermic intraperitoneal chemotherapy, *RECIST* Response Evaluation Criteria in Solid Tumors, *PCI* peritoneal carcinomatosis index, *PRGS* peritoneal regression grading system, *SD* stable disease, *PD* progressive disease

Two patients (17%) ultimately underwent CRS-HIPEC; one was performed by a member of the PIPAC study team, while the second was carried out by an independent treatment team not affiliated with the trial. One patient had a PCI of 17 at the time of CRS-HIPEC; a CC1 cytoreduction was carried out, and there were no surgical complications with a hospital stay of 9 days. The patient started systemic regorafenib within 2 months of surgery. The second patient underwent CRS-HIPEC at an outside institution (operative details not available) and is alive with recurrent disease on systemic therapy at the time of writing. One trial patient who completed the three study PIPACs received five additional treatments (same regimen) on a compassionate use basis over a 9-month period; he is now 1 year out from the last treatment and has PD. He is on oral olaparib and recently started TPN.

**Fig. 4:**
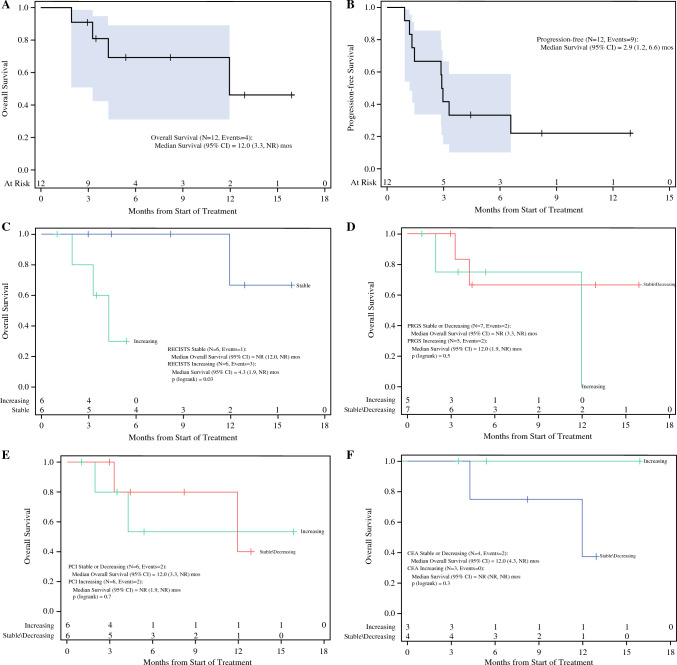
Survival of patients during the trial, and correlation of survival with radiographic, laparoscopic, and histologic response. **A** Overall survival for the cohort was 12.0 months. **B** Progression-free survival was 2.9 months. Correlation of overall survival with clinicopathologic factors including **C** RECIST criteria, **D** PCI, **E** PRGS, or **F** serum CEA level. *RECIST* Response Evaluation Criteria in Solid Tumors, *PCI* peritoneal carcinomatosis index, *PRGS* peritoneal regression grading system

Median OS for patients in the trial was 12.0 months (interquartile range (IQR) 4.3 months to not reached) (Fig. 4A), and median progression-free survival was 2.9 months (IQR 1.4–6.6 months) (Fig. 4B). Stable disease by RECIST criteria was associated with improved overall survival (OS) (median OS not reached) (IQR 12.0 months to not reached) compared with progressive disease (OS 4.3 months; IQR 3.3 months to not reached) (*p* = 0.03; Fig. 4C). However, response by PCI, PRGS, or CEA level was not associated with survival (*p* > 0.1; Fig. 4D–F).

## Discussion

CRC and AC metastatic to the peritoneum is a challenging clinical entity with dismal survival of about 12–15 months with first-line chemotherapy including bevacizumab.^15,16^ In fact, patients with CRC peritoneal metastases have higher risk-adjusted mortality compared with patients with non-peritoneal metastases despite modern chemotherapy regimens.^16^ In patients with resectable disease, CRS with or without HIPEC has been shown to be beneficial,^2,17^ but many patients present with advanced disease not amenable to resection. PIPAC has emerged as a therapeutic modality for IP drug delivery that is performed minimally invasively via laparoscopy and can be administered with repeated procedures for CRC or AC. The current study is the first reported evidence from a phase I trial of PIPAC in the USA for a cohort of patients with unresectable PM who have had progression on at least first-line chemotherapy and therefore have limited therapeutic options and poor prognosis. Compared with prior studies, the current trial enrolled patients who were, on average, more chemotherapy refractory and with higher peritoneal disease burden. In addition, the cohort is more homogeneous than prior studies in that other GI cancers (small bowel, gastric, or hepatobiliary cancers) are not included, allowing for a more accurate interpretation of the efficacy signal. Finally, this study demonstrates from a secondary analysis that radiographic progression correlated with overall survival while evaluation of response using laparoscopic PCI or histologic PRGS changes did not, suggesting response assessment by radiographic criteria as a potential surrogate marker for survival.

The primary endpoints of this phase I trial were feasibility and safety of PIPAC using oxaliplatin at 90 mg/m^2^ dose with and without sensitizing dose of 5-FU and LV chemotherapy. There were no technical failures in this study, demonstrating the feasibility of the procedure and of repeatedly obtaining laparoscopic access to the abdomen. In addition, the majority (58%) of patients in the study were able to undergo more than one PIPAC procedure, and half the patients completed all three, similar to prior the PIPAC-OX trial;^9^ this confirms the feasibility of multiple PIPAC procedures. There were no DLTs or surgical complications in our study. The most common toxicities for oxaliplatin PIPAC are gastrointestinal side effects including abdominal pain, nausea, and vomiting.^8–10^ In our study, most of these toxicities were grade I, with a single grade III toxicity of abdominal pain. There were additional grade III toxicities of anemia, one of which was attributed to 5-FU bolus. Contrary to previous oxaliplatin PIPAC phase I trials, neither pancreatitis^9^ nor greater than grade II neutropenia^8^ were noted. Another distinction from prior studies is that we assessed toxicity of PIPAC oxaliplatin with (cycle 2) and without (cycle 1) sensitizing dose of 5-FU/LV for each patient. While one limitation of this study is the potential for AEs to be related to multiple factors including the disease, surgical procedure, general anesthesia, systemic chemotherapy, or PIPAC IP therapy, care was taken to attribute AEs correctly by discussion amongst all trial investigators at bimonthly meetings using Likert scale of probability per CTCAE convention for systemic chemotherapy trials. This study demonstrated that addition of IV 5-FU/LV within 24 h of PIPAC is safe. Together, these findings confirm not only the feasibility but also the safety and tolerability of multiple cycles of 90 mg/m^2^ oxaliplatin PIPAC with 5-FU/LV systemic chemotherapy.

Our findings of 50% SD by RECIST criteria are also similar to previous trials that showed SD of 62%,^9^ but in our study the median OS and PFS were 12.0 months and 2.9 months, respectively, compared with previously reported OS 4.1 months and PFS 1.5 months in the PIPAC-OX study.^9^ For inclusion in the current study, patients were refractory to at least one line of chemotherapy, but the majority were refractory to two lines of standard-of-care systemic therapy and would therefore historically have limited options for third-line chemotherapy. The OS of 12 months with oxaliplatin PIPAC in conjunction with systemic 5-FU/LV in these patients compares favorably with results from prior trials of third-line systemic chemotherapy. For instance, the median OS with third-line systemic therapy for CRC with PM is noted to be 6.4 months with regorafenib (CORRECT trial^18^) and 7.1 months with TAS-102 (RECOURSE trial^19^). Similarly, the median PFS of oxaliplatin PIPAC with 5-FU/LV in this trial was 2.9 months, while the median PFS was 1.9 months with regorafenib and 2 months with TAS-102.

We noted disease responses with a PCI decrease in 42% of the patients, with another patient (8%) showing stable PCI over the course of the study. Half of the patients had a radiographic response by RECIST criteria, and additionally 58% showed histologic response by PRGS. The most accurate predictor or surrogate of survival in patients undergoing trials of locoregional therapy to the peritoneum is not known. Radiographic measurement via CT scan using RECIST criteria is the mainstay for the evaluation for PM, but CT is limited by image slice thickness (usually 3–5 mm), and meta-analyses have shown sensitivity of 68–83% and specificity of 86–88% for detecting PM on CT.^20,21^ Laparoscopy is used to visualize and quantify PM disease burden, but posttreatment response is limited by scarring and makes calculation of the PCI difficult and subjective. The PRGS^22,23^ has been proposed to evaluate response after locoregional peritoneal therapies and grades the histological appearance of tumor biopsies from 1 (complete response, no tumor cells) to 4 (no response, tumor cells present without any regression). One study did show that cytology and PRGS combined was prognostic for OS and PFS in PM treated with PIPAC.^24^ In our study, secondary analysis of endpoints for response and survival demonstrated correlation of SD by imaging RECIST criteria and OS, but neither response by PCI nor mean PRGS were correlated with OS. PCI is limited in assessment of response because it is difficult to distinguish sclerotic peritoneum after complete tumor regression from confluent tumor. In addition, laparoscopic PCI assessment of response is unable to account for responses that decrease the thickness of the tumor without affecting its size. Similarly, histologic response assessment is subject to sampling error from the biopsies, and owing to prior systemic therapy exposure, most tumor biopsies exhibit some element of response at baseline prior to PIPAC. Another limitation of PRGS is that it can be challenging to make a distinction between intermediate categories of PRGS 2 and 3. The association of RECIST progression with survival is likely because progression on imaging represents a significant increase in the burden of disease that portends a clinically relevant detriment on survival. These findings suggest that response evaluation by RECIST after the initial two PIPACs could be an early surrogate indicator of therapeutic benefit but should be interpreted with caution at this time and validated with additional studies as these findings are a secondary analysis and not the primary outcome of the study. Alternatively, PFS could be a meaningful primary endpoint for a phase II randomized trial.

The current study reports the first results in the USA from a phase I clinical trial of PIPAC in CRC and AC peritoneal carcinomatosis. Oxaliplatin at a dose of 90 mg/m^2^ PIPAC with or without sensitizing 5-FU/LV has demonstrated safety and tolerability over repeated procedures in several clinical trials in Europe, Asia, and now the USA. The current trial results suggest a favorable efficacy profile. Future randomized clinical trials are necessary to prove the efficacy of PIPAC oxaliplatin over standard systemic therapy and specifically evaluate oxaliplatin PIPAC separately in appendiceal and colorectal cancer each.

### Supplementary Information

Below is the link to the electronic supplementary material.

Supplementary file1 (DOCX 49 kb)
