# Supplementary material for: Safety and Efficacy of Oxaliplatin Pressurized Intraperitoneal Aerosolized Chemotherapy (PIPAC) in Colorectal and Appendiceal Cancer with Peritoneal Metastases: Results of a Multicenter Phase I Trial in the USA
Source: Ann Surg Oncol. 2023 Jul 27;30(12):7814–24. doi: 10.1245/s10434-023-13941-2 (PMC10562297; doi:10.1245/s10434-023-13941-2)
Supplement: Supplementary file 1 — Supplementary file1 (DOCX 49 kb) [file 10434_2023_13941_MOESM1_ESM.docx]

**Supplemental Table 1**

| **Adverse Event**  **(n=12)** | **Cycle 1**  **n (%)** | | | | | **Cycle 2**  **n (%)** | | | | |
| --- | --- | --- | --- | --- | --- | --- | --- | --- | --- | --- |
|  | **Grade 1** | **Grade 2** | **Grade 3** | **Grade 4** | **Grade 5** | **Grade 1** | **Grade 2** | **Grade 3** | **Grade 4** | **Grade 5** |
| ABDOMINAL PAIN | 3(25%) | 1(8%) | --- | --- | --- | 1(8%) | --- | 1(8%) | --- | --- |
| ANEMIA | 1(8%) | --- | --- | --- | --- | --- | --- | 1(8%) | --- | --- |
| NAUSEA\VOMITING | 4(33%) | 1(8%) | --- | --- | --- | --- | --- | --- | --- | --- |
| CONSTIPATION | 3(25%) | 1(8%) | --- | --- | --- | 1(8%) | --- | --- | --- | --- |
| FATIGUE | 1(8%) | 1(8%) | --- | --- | --- | 1(8%) | --- | --- | --- | --- |
| HYPOPHOSPHATEMIA | --- | 1(8%) | --- | --- | --- | --- | --- | --- | --- | --- |
| HYPOTENSION | --- | 1(8%) | --- | --- | --- | --- | --- | --- | --- | --- |
| ILEUS | --- | 1(8%) | --- | --- | --- | --- | --- | --- | --- | --- |
| PLATELET COUNT DECREASED | --- | 1(8%) | --- | --- | --- | 1(8%) | --- | --- | --- | --- |
| ABDOMINAL DISTENSION | 3(25%) | --- | --- | --- | --- | --- | --- | --- | --- | --- |
| ANOREXIA | 2(17%) | --- | --- | --- | --- | --- | --- | --- | --- | --- |
| DIARREHA | 2(17%) | --- | --- | --- | --- | --- | --- | --- | --- | --- |
| DIZZINESS | 1(8%) | --- | --- | --- | --- | --- | --- | --- | --- | --- |
| GENERALIZED MUSCLE WEAKNESS | 1(8%) | --- | --- | --- | --- | --- | --- | --- | --- | --- |
| HYPERNATREMIA | 1(8%) | --- | --- | --- | --- | --- | --- | --- | --- | --- |
| HYPOALBUMINEMIA | 1(8%) | --- | --- | --- | --- | --- | --- | --- | --- | --- |
| HYPOCALCEMIA | 1(8%) | --- | --- | --- | --- | --- | --- | --- | --- | --- |
| HYPOKALEMIA | 1(8%) | --- | --- | --- | --- | --- | --- | --- | --- | --- |
| HYPONATREMIA | 1(8%) | --- | --- | --- | --- | --- | --- | --- | --- | --- |
| MUSCLE CRAMP | 1(8%) | --- | --- | --- | --- | --- | --- | --- | --- | --- |
| NON-CARDIAC CHEST PAIN | 1(8%) | --- | --- | --- | --- | --- | --- | --- | --- | --- |
| URINE OUTPUT DECREASED | 1(8%) | --- | --- | --- | --- | --- | --- | --- | --- | --- |
| WHITE BLOOD CELL COUNT DECREASED | 1(8%) | --- | --- | --- | --- | 1(8%) | --- | --- | --- | --- |

**Supplemental Table 2**

| Patient | Cycle # | Cmax (µg/mL) | AUC0-t (µgxhr/mL) |
| --- | --- | --- | --- |
| 001 | 1 | 2.99 | 42.95 |
|  | 2 | 4.63 | 101.77 |
|  | 3 | 4.10 | 72.16 |
| 004 | 1 | 0.88 | 18.12 |
|  | 2 | 3.91 | 59.00 |
|  | 3 | 1.69 | 29.27 |
| 006 | 1 | 0.99 | 20.94 |
| 007 | 1 | 2.57 | 42.75 |
|  | 2 | 2.83 | 45.66 |
|  | 3 | 3.22 | 49.61 |
| 008 | 1 | 2.69 | 38.02 |
| 015 | 1 | 1.11 | 20.25 |
|  | 2 | 1.56 | 31.08 |

**Supplemental Table 3**

| Patient | PIPAC Cycle 1 | | PIPAC Cycle 2 | | PIPAC Cycle 3 | |
| --- | --- | --- | --- | --- | --- | --- |
|  | Best PRGS | Mean PRGS | Best PRGS | Mean PRGS | Best PRGS | Mean PRGS |
| 1 | 2 | 2.5 |  |  |  |  |
| 2 | 2 | 2.7 | 2 | 2.3 | 2 | 2.0 |
| 3 | 2 | 2.8 | 2 | 2.3 |  |  |
| 4 | 2 | 2.0 | 2 | 2.5 | 2 | 3.0 |
| 5 | 2 | 2.0 | 1 | 1.5 |  |  |
| 6 | 2 | 2.8 | 3 | 3.0 | 2 | 2.5 |
| 7 | 2 | 2.0 | 2 | 2.0 | 2 | 2.0 |
| 8 | 1 | 2.0 |  |  |  |  |
| 9 | 2 | 2.5 | 2 | 2.8 |  |  |
| 10 | 2 | 2.5 | 2 | 2.8 |  |  |
| 11 | 2 | 2.5 | 2 | 2.5 | 1 | 2.5 |
| 12 | 2 | 2.5 | 2 | 2.5 | 2 | 2.3 |

PRGS, peritoneal regression grading score.

**Supplemental Figure 1**
